# Gastrointestinal Symptoms in Celiac Disease Patients on a Long-Term Gluten-Free Diet

**DOI:** 10.3390/nu8070429

**Published:** 2016-07-14

**Authors:** Pilvi Laurikka, Teea Salmi, Pekka Collin, Heini Huhtala, Markku Mäki, Katri Kaukinen, Kalle Kurppa

**Affiliations:** 1 School of Medicine, University of Tampere, Tampere 33014, Finland; laurikka.pilvi.l@student.uta.fi (P.L.); teea.salmi@uta.fi (T.S.); pekka.collin@uta.fi (P.C.); markku.maki@uta.fi (K.K.); 2 Department of Dermatology, Tampere University Hospital, Tampere 33014, Finland; 3 Department of Gastroenterology and Alimentary Tract Surgery, Tampere University Hospital, University of Tampere, Tampere 33014, Finland; 4 Tampere School of Health Sciences, University of Tampere, Tampere 33014, Finland; heini.huhtala@staff.uta.fi; 5 Centre for Child Health Research, University of Tampere and Tampere University Hospital, Tampere 33014, Finland; markku.maki@uta.fi; 6 Department of Internal Medicine, Tampere University Hospital, Tampere 33014, Finland

**Keywords:** celiac disease, gastrointestinal diseases, symptoms, gluten-free diet

## Abstract

Experience suggests that many celiac patients suffer from persistent symptoms despite a long-term gluten-free diet (GFD). We investigated the prevalence and severity of these symptoms in patients with variable duration of GFD. Altogether, 856 patients were classified into untreated (*n* = 128), short-term GFD (1–2 years, *n* = 93) and long-term GFD (≥3 years, *n* = 635) groups. Analyses were made of clinical and histological data and dietary adherence. Symptoms were evaluated by the validated GSRS questionnaire. One-hundred-sixty healthy subjects comprised the control group. Further, the severity of symptoms was compared with that in peptic ulcer, reflux disease, inflammatory bowel disease (IBD) and irritable bowel syndrome (IBS). Altogether, 93% of the short-term and 94% of the long-term treated patients had a strict GFD and recovered mucosa. Untreated patients had more diarrhea, indigestion and abdominal pain than those on GFD and controls. There were no differences in symptoms between the short- and long-term GFD groups, but both yielded poorer GSRS total score than controls (*p* = 0.03 and *p* = 0.05, respectively). Furthermore, patients treated 1–2 years had more diarrhea (*p* = 0.03) and those treated >10 years more reflux (*p* = 0.04) than controls. Long-term treated celiac patients showed relatively mild symptoms compared with other gastrointestinal diseases. Based on our results, good response to GFD sustained in long-term follow-up, but not all patients reach the level of healthy individuals.

## 1. Introduction

The only current treatment for celiac disease is a life-long gluten-free diet. Commencement of a strict diet usually results in prompt relief of clinical symptoms, while recovery of small-bowel mucosal damage may take even years [1,2]. Although mucosal healing is the ultimate goal of the dietary treatment [3], from the patient’s perspective alleviation of self-perceived clinical symptoms is usually the most rewarding outcome. A good clinical response in the early stages of dietary treatment further motivates to maintain a strict diet, which consequently facilitates mucosal recovery. There is some evidence that after the initial enthusiasm has faded, many patients experience ongoing symptoms while maintaining an apparently strict gluten-free diet [4,5,6,7]. Such persistence of symptoms despite burdensome dietary restriction is frustrating and may even predispose to poor dietary adherence and thus further worsen the situation. Hitherto, however, neither the prevalence nor the severity of the persistent symptoms in celiac disease patients on a gluten-free diet has been well characterized, let alone their impact on patients’ daily life. Data on these aspects would be necessary in order to optimize the follow-up of patients and, in the future, to develop interventions on top of the gluten-free diet.

The aim of the present nationwide study was to define the prevalence and severity of gastrointestinal symptoms in a large cohort of long-term dietary treated adult celiac disease patients and to compare these with those seen in untreated and short-term treated patients and in healthy controls. Further, symptom severity was compared with other common gastrointestinal diseases based on a literature search.

## 2. Materials and Methods

### 2.1. Study Design and Participants

The large cross-sectional study was carried out at Tampere University Hospital and the University of Tampere. The celiac disease patients were collected from our prospectively maintained research database, in which the patients have been recruited via newspaper advertisements and via local and national celiac disease associations from different parts of Finland. Exclusion criteria for the present study were age under 15 years and uncertain diagnosis of celiac disease (not based on biopsy). The final study cohort comprised 856 consecutive subjects with confirmed celiac disease. All celiac disease patients had received professional dietary counseling and were placed on a strict gluten-free diet soon after the diagnosis was confirmed.

Clinical data were collected systematically from the medical records. Further, all study subjects were interviewed by an experienced physician or a study nurse in the study clinic and asked about demographics, clinical presentation of the disease at the time of diagnosis, family history of celiac disease, duration of gluten-free diet, and adherence to dietary treatment. The main mode of presentation of celiac disease at diagnosis was further classified into gastrointestinal symptoms (e.g., indigestion, diarrhea and signs of malabsorption), extraintestinal symptoms (e.g., dermatitis herpetiformis, dental enamel defects and neurological symptoms) and patients detected by screening at-risk groups (celiac disease in family, type I diabetes, thyroid disease, Sjögren’s syndrome, Addison’s disease and IgA nephropathy). The results of serum endomysial antibody (EmA) measurements and small-bowel mucosal biopsy sampling were collected systematically.

In order to compare differences in the presence and severity of persistent gastrointestinal symptoms between subjects dieting for different periods of gluten-free diet the celiac disease patients were further divided into three groups based on the duration of the gluten-free diet as follows: (i) newly diagnosed patients (no diet); (ii) patients with short-term treatment (diet 1–2 years) and (iii) patients with long-term treatment (diet ≥ 3 years). For a more detailed analysis the long-term treatment group was further divided into subjects who had been on a gluten-free diet either 3–5 years, 6–10 years or > 10 years.

One-hundred-and-sixty healthy individuals (72% females, median age 55 (range 23–87) years) with no first-degree relatives with celiac disease served as a control group in the comparison of gastrointestinal symptoms.

The Regional Ethics Committee of the Tampere University Hospital District approved the study protocol and all participants gave written informed consent.

### 2.2. Celiac Disease Serology and Small-Bowel Mucosal Histology

Serum IgA class EmA were measured by an indirect immunofluorescence method with human umbilical cord as substrate [8], and a dilution of 1: ≥5 was considered positive. Positive samples were further diluted up to 1:4000 until negative. In cases of selective IgA deficiency, the corresponding IgG class antibodies were measured. Serum transglutaminase 2 antibodies had also been measured in most of the patients, but since test methods and reference values had varied during the study period these readings were not used here. The degree of small-bowel mucosal damage was systematically measured from several well-orientated duodenal biopsy samples and reported by quantitative villous height crypt depth ratio (VH/CrD) as previously described [9]. Here VH/CrD < 2.0 was considered to indicate active celiac disease [9].

### 2.3. Gastrointestinal Symptoms 

For the systematic evaluation of current gastrointestinal symptoms, participants in each group filled a self-administered, structured Gastrointestinal Symptom Rating Scale (GSRS) questionnaire. This is a validated questionnaire used widely in research on celiac disease and other gastrointestinal disorders [10,11,12,13,14,15]. The questionnaire measures five sub-dimensions of gastrointestinal symptoms: Indigestion, diarrhea, abdominal pain, reflux and constipation. It comprises altogether 15 separate items. Values for each of the five sub-dimension scores were calculated as a mean of the respective items and the total GSRS score as a mean of all 15 items. The scoring is based on a Likert scale from 1 to 7 points, where 1 point signifies minimal gastrointestinal symptoms and 7 points the most severe symptoms.

To further identify patients with persistent gastrointestinal symptoms, a cut-off for significantly worsened symptoms was set in the GSRS total and sub-dimension scores. This was the case when the subject’s GSRS total score or subscore was higher than 1 standard deviation (SD) compared with the corresponding mean score or subscore of the healthy controls [4,16,17,18].

Besides between study groups, the GSRS scores in untreated and long-term treated celiac disease patients were compared with those seen in subjects with common gastrointestinal disorders, namely peptic ulcer, gastro-esophageal reflux disease (GER), inflammatory bowel disease (IBD) and irritable bowel syndrome (IBS) as established by literature search [11,13,14,15]. In all diseases the GSRS scores were from untreated patients at diagnosis except for IBD, where the subjects were on treatment.

### 2.4. Adherence to the Gluten-Free Diet

Based on dietary interview, a subject was considered to be adherent to the gluten-free diet in the case of minor inadvertent gluten intake a few times a year or less. In addition, an objective estimation of dietary adherence was carried out by measuring the percentage of EmA-positive subjects in each treatment group. Positivity for EmA was considered to represent non-adherence when detected after two years on a gluten-free diet [19].

### 2.5. Statistics

Categorical data were described using percentages and quantitative data using either medians with range or means with 95% confidence intervals. Cross-tabulation with Pearson’s χ2 test was used to analyze differences between categorical variables. To compare means between study groups, one-way ANOVA with Bonferroni post hoc analysis was used in normally distributed variables and Kruskal-Wallis test in non-parametric variables. To investigate correlation between variables, correlation coefficient (r) was calculated using Spearman’s correlation. To take account of the effect of age, a covariance analysis was used. Analyses were made with the whole study cohort and also separately for males and females. A *p*-value < 0.05 was considered statistically significant. All data were analyzed by SPSS (Statistical Package for the Social Sciences) Statistics Version 21 (IBM Corporation, Armonk, NY, USA).

## 3. Results

The median age of all 856 celiac disease patients was 54 years (range 15–85 years) and 75% were females. In 64% the reasons for celiac disease suspicion were gastrointestinal symptoms and in 18% extraintestinal symptoms; 18% were detected by screening. There were no significant differences between the celiac disease groups in either gender, median age at time of study, clinical presentation at diagnosis or celiac disease in the family (Table 1). Among patients on a gluten-free diet the long-term treated cohort contained lower percentage of EmA-positive subjects than the short-term treated, while there were no differences in self-reported dietary adherence or VH/CrD.

**Figure 1:**
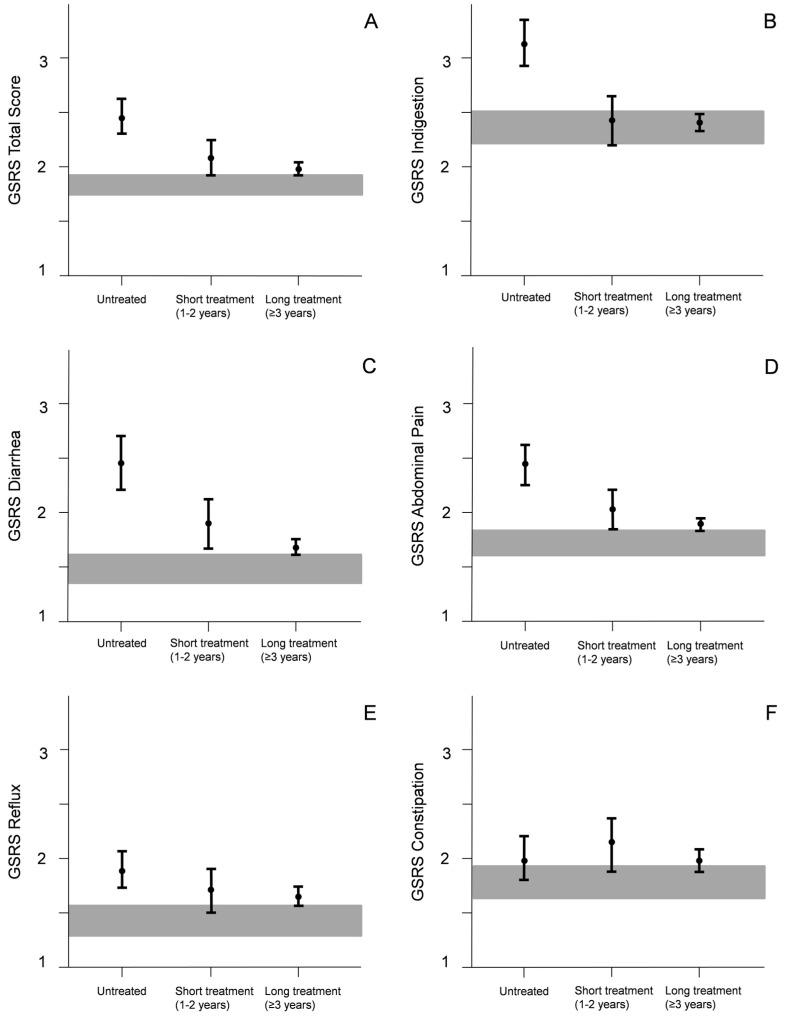
Gastrointestinal Symptom Rating Scale (GSRS) total (**A**) and sub-dimension (**B**–**F**) scores in untreated, short-term treated and long-term treated patients compared with healthy controls. Values are expressed as means with 95% confidence intervals (CI) and gray bars denote 95% CIs of controls. There were significant differences between the groups as follows: (**A**) Untreated patients and all other groups (*p* < 0.001), and short-term treated patients and healthy controls (*p* = 0.03); (**B**) Untreated and all other groups (*p* < 0.001); (**C**) Untreated and short-term treated (*p* = 0.015) and long-term treated (*p* < 0.001) patients, and short-term treated patients and healthy controls (*p* = 0.010); (**D**) Untreated patients and all other groups (*p* < 0.001); (**E**) Healthy controls and untreated (*p* < 0.001) and long-term treated (*p* = 0.013) patients.

Untreated celiac patients had significantly higher (more symptoms) GSRS scores on indigestion, diarrhea, abdominal pain and total scores than those on a gluten-free diet and healthy controls, whereas there was no difference in any of the scores between long-term and short-term treated patients (Figure 1). Long-term treated patients yielded higher GSRS reflux scores, and short-term treated higher diarrhea and total scores compared with the healthy controls. In more detailed analysis reflux was seen particularly in patients treated >10 years (data not shown). None of the gastrointestinal symptoms correlated with VH/CrD levels either in the whole cohort (r varying between −0.013 and −0.220) or in the different durations of gluten-free diet (r varying between −0.146 and 0.142).

When analyzing the occurrence of significantly increased (by definition, GSRS score > 1 SD compared with healthy controls) gastrointestinal symptoms, the untreated celiac disease patients again showed significant overrepresentation in all GSRS scores except constipation compared with the other study groups (Table 2). In addition, both long- and short-term treatment groups evinced more reflux and total gastrointestinal symptoms than controls; the short-term treated patients also reported more diarrhea (Table 2). In treatment groups the mean VH/CrD levels did not differ between patients with and without increased symptoms in any of the GSRS sub-groups (data not shown). In separate analysis, long-term treated women had higher GSRS total scores (2.0 vs. 1.8, *p* = 0.001) and indigestion scores (2.5 vs. 2.3, *p* = 0.034) than men, whereas short-term treated men had higher diarrhea scores (2.4 vs. 1.7, *p* = 0.003). The overrepresentation of increased (>1 SD) reflux and total scores seen in the long-term treatment group in both genders combined remained significant in women (*p* = 0.002 and *p* = 0.015, respectively) but not in men. The GSRS diarrhea scores were also increased in short-term treated men and long-term treated women compared with healthy controls (2.4 vs. 1.5, *p* < 0.001 and 1.7 vs. 1.5, *p* = 0.042, respectively). 

**Figure 2:**
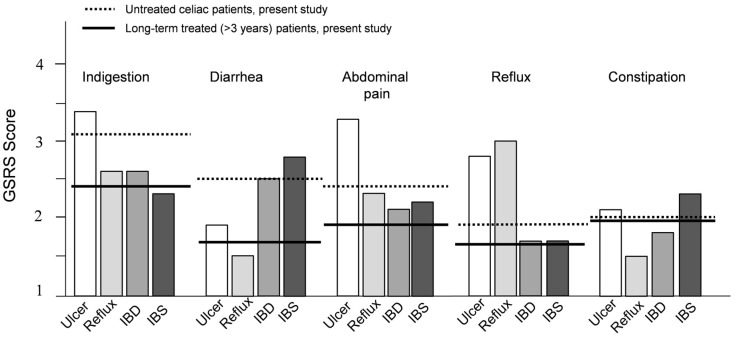
The mean Gastrointestinal Symptom Rating Scale (GSRS) sub-dimension scores of untreated and long-term treated patients (present study) compared with other gastrointestinal diseases [11,13,14,15]. Ulcer, peptic ulcer disease; Reflux, gastroesophageal reflux disease; IBD, inflammatory bowel disease; IBS, irritable bowel syndrome.

Comparisons of GSRS scores between celiac disease patients in the present study (untreated and long-term treated) and those with other gastrointestinal disorders can be seen in Figure 2. In general, untreated celiac patients suffered from a wider spectrum of symptoms compared with other gastrointestinal disease groups, the most severe being indigestion, diarrhea and abdominal pain (Figure 2). However, in long-term treated patients the gastrointestinal symptoms were clearly milder.

## 4. Discussion

The main finding in the present study was that both short-term and long-term dietary treated celiac disease patients have more symptoms than non-celiac controls. However, although the majority of gastrointestinal symptoms are alleviated well on a strict gluten-free diet, not all patients reach the level of the general population even in long-term follow-up.

Here, the majority of the celiac disease patients showed rapid relief of symptoms during the first year on a gluten-free diet. This is in accord with previous studies investigating short-term responses, where the diet has also alleviated typical gastrointestinal symptoms within the first few months after diagnosis [20,21,22]. The only exception here was diarrhea, which, although alleviated on a long-term diet, remained fairly common in short-term-treated patients. This raises the question whether the alleviation of diarrhea requires more complete histological recovery than other symptoms. However, in a recent study we observed no differences in symptoms or quality of life between patients evincing full histological recovery and those with ongoing mucosal damage after one year [22]. Hence, incomplete mucosal recovery would not appear to explain slow recovery from diarrhea in our patients. The few previous studies investigating this issue have obtained somewhat contradictory results. In accord with our observations, Pulido and colleagues showed very slow resolution of diarrhea on treatment within five or more years [7], whereas a group under Murray observed improvement of diarrhea already within six months [20]. The reason for these considerable variations between the studies remains unclear, but might for example involve differences in interpretation and definition of symptoms. Obviously different study designs and populations may also have an effect, as we had demographic characteristics and design similar to those of Pulido and colleagues [7], whereas Murray and group [20] investigated mainly short-term responses to a gluten-free diet in one well-defined geographical region.

In contrast to the well-documented short-term outcome [2], the long-term response to a gluten-free diet has thus far been poorly investigated. Judging from our results, in most patients with good adherence and recovered villi the good initial response to the diet remains after several years, demonstrating that it is not only based on a short-term “honeymoon” effect. Notwithstanding this long-lasting positive effect, we found even long-term dietary treated patients to have more symptoms than healthy controls. Such ongoing symptoms may in the long run discourage patients from adhering to what is a socially restrictive and expensive treatment mode if they consider it ineffective. In such cases, it is particularly important for physicians to urge patients to persist with a strict gluten-free diet in order to prevent disease-associated complications [2]. In addition to the increased GSRS total score, particularly reflux symptoms showed a tendency to persist for several years. Gastroesophageal reflux is common in general populations [23] and in earlier studies it has appeared to be approximately as common in celiac patients [24]. Then again, initiation of a gluten-free diet has often reduced reflux symptoms rapidly [25,26,27], and the response has also persisted in the long term [27]. In some studies reflux symptoms have been suggested to be more common in aged people [28], but this is controversial and did not explain the difference in the present study. Altogether, the reason for the increase in reflux symptoms in long-term treated celiac disease remains unclear and needs to be clarified in future studies.

We observed long-term treated celiac disease women to experience more symptoms than men. Previously Hallert and associates has reported similar findings in Swedish women [6,29,30], and Pulido and colleagues observed more symptoms in both undiagnosed Canadian women and those on dietary treatment [7]. One plausible explanation for the gender difference might be the higher prevalence of concomitant functional gastrointestinal disorders in women, which have also been shown to be exacerbated by psychological distress such as that involved in following a burdensome dietary treatment [31,32,33]. Women may also find the inevitable social restrictions caused by the gluten-free diet harder to cope with [29]. Other possible reasons could be differences in fiber intake and the symptom-modifying effect of gonadal hormones [34,35,36]. In any case, physicians should acknowledge the higher risk of persistent symptoms in women and provide adequate support if needed.

In comparison with the other common gastrointestinal disorders as reported in the literature [11,13,14,15], we observed that untreated celiac patients evince a fairly wide range of symptoms. In line with this, other recent studies have reported that nowadays only a minority of patients present with classical symptoms, such as diarrhea and malabsorption, but, instead, suffer from a plethora of “atypical” symptoms or have no symptoms at all [4,19,37]. As well as in the diagnostic workout, this heterogeneous clinical presentation also needs to be taken into account when evaluating the long-term dietary response. In particular, although the specific GSRS scores here were mostly fairly low, physicians should remember that suffering from multiple, even if moderate, symptoms simultaneously may constitute a substantial burden in individual patients.

The most common reason for the persistence of symptoms in celiac disease has been ongoing gluten consumption [5,38]. However, in agreement with our previous studies [19,39,40], more than 90% of the patients here were strictly adherent and even in the few who reported lapses these were only occasional. This conception was further confirmed by the well-recovered histology and low EmA-positivity among patients on the diet. Thus, although gluten intake should always be excluded [41], other explanations for persistent symptoms must be sought in patients with proven strict adherence. These include for example small-intestinal bacterial overgrowth or some other concomitant disorder such as IBD and microscopic colitis, and refractory celiac disease [5,41]. An interesting new research topic related to this issue is dysbiosis of the intestinal microbiota [42]. We have recently shown that celiac patients suffering from persistent symptoms on a gluten-free diet had an altered balance and reduced richness of duodenal microbiota [43]. The intestinal microbiota affects the complex gut-brain axis along with the enteric nervous system, immune system and external environment, and alterations in this axis may predispose to chronic pain in functional gastrointestinal disorders and perhaps also in celiac disease [44]. A deeper understanding of these mechanisms would be important in order to make the development of new pharmacological interventions possible.

Several novel adjunct therapies to improve the treatment of celiac disease are currently under development [45]. In these circumstances evaluating long-term symptoms in treated celiac disease patients becomes more and more important. For now, gluten-free diet remains the gold standard treatment for celiac disease and thus every new therapeutic approach needs to be compared with the response to the diet. The present study provides solid information of the response of celiac individuals to gluten-free diet and could thus be used as baseline to the future pharmacological trials.

Strengths of the present study were the large and nationwide cohort of clinically representative celiac disease patients and the use of well-validated and structured symptom questionnaire. A major limitation was the retrospective cross-sectional study design, this, however, being offset by the fact that patients with different durations of gluten-free diet were comparable regarding most of the clinical and demographic parameters and were diagnosed and treated similarly according to nationwide guidelines. Another limitation was that the majority of the participants were recruited via celiac disease associations, which may cause some selection bias. Finally, celiac disease was not excluded among all healthy controls, a few of whom might have been suffering unrecognized.

## 5. Conclusions

In conclusion, we showed that the good initial clinical response to a gluten-free diet is sustained also in the long run. However, it is important for physicians to realize that one year might not be long enough for all symptoms to abate, and that some patients may continue to have mild or moderate gastrointestinal symptoms despite long-term and strict dietary treatment. A fuller understanding of the factors behind persistent symptoms in celiac disease would provide new treatment possibilities in the future.

## Figures and Tables

**Table 1:** Demographic characteristics and selected celiac disease-associated data on untreated, short-term (1–2 years) treated and long-term (≥3 years) treated celiac patients and healthy controls.

		Celiac Patients on a GFD *n* = 728		
	Untreated Patients *n* = 128	Short Treatment *n* = 93	Long Treatment *n* = 635	Non-Celiac Controls *n* = 160
Females, %	76	72	75	72
Current age, median (range)	47 (15–72)	51 (16–80)	55 (17–85)	55 (23–87)
GFD, median (range), years.	0	1 (1–2)	12 (3–48)	0
Mode of presentation at diagnosis, %				
Gastrointestinal	66	63	64	0
Extraintestinal ^a^	12	16	19	0
Screen-detected ^b^	23	20	17	0
Celiac disease in family, %	47	54	61	0
Self-reported strictness of GFD, (%)				
Strict diet	0	93	94	0
Occasional gluten	0	7	6	0
No diet	100	0	0	100
Positive EMA, %	93	8 ^c^	3 ^c,d^	0 ^e^
VH/CrD, mean (95% CI)	0.5 (0.4–0.6)	2.7 (2.5–2.9) ^c,f^	2.8 (2.6–2.9) ^c,g^	3.2 (3.0–3.3) ^h^

^a^ Dermatitis herpetiformis, aphtous ulcerations, enamel defects, elevated liver enzymes, neurological and musculoskeletal symptoms, psychiatric symptoms, infertility or early menopause; ^b^ Family history of celiac disease, type I diabetes, thyroidal disease, Sjögren’s syndrome, Addison’s disease, IgA nephropathy; ^c^ *p* < 0.001 compared with untreated patients; ^d^ *p* = 0.028 compared with short treatment group; ^e–h^ Data available on ^e^ 50 subjects, ^f^ 20 subjects, ^g^ 191 subjects and ^h^ 35 subjects. GFD, gluten-free diet; EMA, endomysial antibodies; VH/CrD, small-bowel mucosal villous height crypt depth ratio; CI, confidence interval.

**Table 2:** Presence (%) of increased gastrointestinal symptoms^a^ in untreated, short-term (GFD 1–2 years) treated and long-term (GFD ≥ 3 years) treated celiac disease patients and in healthy controls.

		Celiac Patients on a GFD *n* = 728		
GSRS Score	Untreated Patients *n* = 128	Short Treatment *n* = 93	Long Treatment *n* = 635	Non-Celiac Controls *n* = 160
Total score	48 ^b^	27 ^c^	23 ^c^	16
Indigestion	41 ^b^	18	17	14
Diarrhea	47 ^b^	32 ^c,d^	21	15
Abdominal pain	43 ^b^	20	18	14
Reflux	34 ^b^	20 ^c^	19 ^c^	11
Constipation	16	18	18	16

GSRS, Gastrointestinal Symptom Rating Scale; GFD, gluten-free diet. ^a^ Defined as GSRS scores > 1 SD compared to mean values of healthy controls; ^b^ *p* < 0.05 compared with short and long treatment groups and with controls; ^c^ *p* < 0.05 compared with healthy controls; ^d^ *p* < 0.05 compared with long treatment group.
